# Comparative molecular profiling of multidrug-resistant *Pseudomonas aeruginosa* identifies novel mutations in regional clinical isolates from South India

**DOI:** 10.1093/jacamr/dlae001

**Published:** 2024-01-16

**Authors:** Nitasha D Menon, Priyanka Somanath, Jennifer Jossart, Gayathri Vijayakumar, Kavya Shetty, Manasi Baswe, Meghna Chatterjee, Malavika B Hari, Samitha Nair, V Anil Kumar, Bipin G Nair, Victor Nizet, J Jefferson P Perry, Geetha B Kumar

**Affiliations:** School of Biotechnology, Amrita Vishwa Vidyapeetham, Amritapuri, Kerala, India; Antimicrobial Resistance, Tata Institute for Genetics and Society (TIGS), Bangalore, India; School of Biotechnology, Amrita Vishwa Vidyapeetham, Amritapuri, Kerala, India; Antimicrobial Resistance, Tata Institute for Genetics and Society (TIGS), Bangalore, India; Department of Molecular Diagnostics and Experimental Therapeutics, City of Hope, Duarte, CA, USA; School of Biotechnology, Amrita Vishwa Vidyapeetham, Amritapuri, Kerala, India; Antimicrobial Resistance, Tata Institute for Genetics and Society (TIGS), Bangalore, India; School of Biotechnology, Amrita Vishwa Vidyapeetham, Amritapuri, Kerala, India; Antimicrobial Resistance, Tata Institute for Genetics and Society (TIGS), Bangalore, India; School of Biotechnology, Amrita Vishwa Vidyapeetham, Amritapuri, Kerala, India; Antimicrobial Resistance, Tata Institute for Genetics and Society (TIGS), Bangalore, India; School of Biotechnology, Amrita Vishwa Vidyapeetham, Amritapuri, Kerala, India; Antimicrobial Resistance, Tata Institute for Genetics and Society (TIGS), Bangalore, India; School of Biotechnology, Amrita Vishwa Vidyapeetham, Amritapuri, Kerala, India; Antimicrobial Resistance, Tata Institute for Genetics and Society (TIGS), Bangalore, India; Department of Microbiology, DDRC SRL Diagnostic Private Limited, Trivandrum, Kerala, India; Department of Microbiology, Amrita Institute of Medical Sciences, Amrita Vishwa Vidyapeetham, Kochi, Kerala, India; School of Biotechnology, Amrita Vishwa Vidyapeetham, Amritapuri, Kerala, India; Antimicrobial Resistance, Tata Institute for Genetics and Society (TIGS), Bangalore, India; Department of Pharmacology, University of California, San Diego, La Jolla, CA, USA; Skaggs School of Pharmacy and Pharmaceutical Sciences, University of California, San Diego, La Jolla, CA, USA; Department of Pediatrics, University of California, San Diego, La Jolla, CA, USA; Department of Molecular Diagnostics and Experimental Therapeutics, City of Hope, Duarte, CA, USA; School of Biotechnology, Amrita Vishwa Vidyapeetham, Amritapuri, Kerala, India; Antimicrobial Resistance, Tata Institute for Genetics and Society (TIGS), Bangalore, India

## Abstract

**Objectives:**

We sought to analyse the antibiotic susceptibility profiles and molecular epidemiology of MDR clinical *Pseudomonas aeruginosa* isolates from South India using non-MDR isolates as a reference.

**Methods:**

We established a comprehensive clinical strain library consisting of 58 isolates collected from patients across the South Indian state of Kerala from March 2017 to July 2019. The strains were subject to antibiotic susceptibility testing, modified carbapenem inactivation method assay for carbapenemase production, PCR sequencing, comparative sequence analysis and quantitative PCR of MDR determinants associated with antibiotic efflux pump systems, fluoroquinolone resistance and carbapenem resistance. We performed *in silico* modelling of MDR-specific SNPs.

**Results:**

Of our collection of South Indian *P. aeruginosa* clinical isolates, 74.1% were MDR and 55.8% were resistant to the entire panel of antibiotics tested. All MDR isolates were resistant to levofloxacin and 93% were resistant to meropenem. We identified seven distinct, MDR-specific mutations in *nalD*, three of which are novel. *mexA* was significantly overexpressed in strains that were resistant to the entire test antibiotic panel while *gyrA* and *gyrB* were overexpressed in MDR isolates. Mutations in fluoroquinolone determinants were significantly associated with MDR phenotype and a novel GyrA Y100C substitution was observed. Carbapenem resistance in MDR isolates was associated with loss-of-function mutations in *oprD* and high prevalence of NDM (*bla*_NDM-1_) within our sample.

**Conclusions:**

This study provides insight into MDR mechanisms adopted by *P. aeruginosa* clinical isolates, which may guide the potential development of therapeutic regimens to improve clinical outcomes.

## Introduction

The rapid emergence of MDR, XDR and pandrug-resistant (PDR) strains leaves clinicians few effective treatment options for several important bacterial infections. In 2019, antimicrobial resistance (AMR) was associated with approximately 4.95 million deaths worldwide, with 1.39 million of these infections concentrated in South Asia.^1^ This region accounts for approximately a quarter of global antibiotic consumption and the COVID-19 pandemic has further exacerbated this AMR crisis.^2–4^ A better understanding of the molecular determinants responsible for the resistance phenotypes can provide better insight on treatment modalities as well as shape alternative strategies for combating the global crisis of AMR.

*Pseudomonas aeruginosa* is a nosocomial pathogen commonly associated with MDR ventilator-associated pneumonia (VAP), catheter-associated urinary tract infection (CAUTI), wound or burn infections, septicaemia and diabetic foot infections, particularly in immunocompromised individuals.^5–7^  *P. aeruginosa,* a WHO-designated critical priority pathogen is intrinsically resistant to many antimicrobial agents, and has a dynamic, plastic genome that can acquire resistance to virtually all commercially available antibiotics through multiple mechanisms including the presence of antibiotic efflux pumps (e.g. Mex RND family systems) or loss of antibiotic import porins (e.g. OprD carbapenem entry porin), alteration in cellular targets of antibiotics (e.g. *gyrAB* or *parEC* mutations in fluoroquinolone resistance) and production of specific enzymes that inactivate antibiotics (e.g. β-lactamases or carbapenemases).^8,9^

While there is existing literature on resistance rates, there is limited information on the prevalence of genetic determinants and molecular mechanisms of resistance among clinical *P. aeruginosa* strains, particularly from South India.^10–14^ Furthermore, many studies lack analysis of non-MDR, antibiotic-susceptible strains as a reference. In this study, we conducted AMR profiling of 58 clinical *P. aeruginosa* isolates from patients in the southern Indian state of Kerala. By comparing MDR and non-MDR isolates, we investigated the molecular mechanisms underlying resistance and their frequencies in the region. Our goal is to contribute to a broader understanding of MDR and carbapenem-resistant *P. aeruginosa* in India, providing insights for combating AMR at both regional and global levels.

## Materials and methods

### Bacterial isolate collection and culturing conditions

Convenience sampling of isolates for the clinical strain library was performed by obtaining strains from patients at healthcare centres and diagnostic laboratories located across the state of Kerala, India as part of their routine care from March 2017 to July 2019. Bacterial growth conditions were performed as previously described.^15^

### Antibiotic susceptibility testing

MIC was determined using a fully automated VITEK 2 system (bioMérieux, Inc., Hazelwood, MO, USA) as per the manufacturer’s instructions and further confirmed using disc diffusion testing.^15,16^ Antibiotics tested include piperacillin/tazobactam, ceftazidime, cefoperazone/sulbactam, cefepime, imipenem, meropenem, amikacin, gentamicin, ciprofloxacin and levofloxacin. Strains were defined as MDR if they were resistant to three or more classes of antibiotics.

### Modified carbapenem inactivation method (mCIM)

mCIM assays were performed for detection of carbapenemase production as previously described.^17^

### Bacterial DNA isolation, PCR and Sanger sequencing

DNA was isolated from stationary-phase cultures using the DNeasy UltraClean Microbial Kit (QIAGEN, 12224-50). PCR amplification of AMR genes and Sanger sequencing were performed using the primer sets listed in Table S1 (available as Supplementary data at *JAC-AMR* Online) for *mexR*, *nalD*, *mexS*, *mexT*, *gyrA*, *gyrB*, *parC*, *parE*, *oprD* and *bla*_NDM-1_. Amplicons were generated using Q5 High Fidelity DNA polymerase (New England Biosciences, M0491) as per the manufacturer’s protocol in an S1000 Thermal Cycler (Bio-Rad). Sanger sequencing was performed at the sequencing facility at the National Centre for Biological Sciences (NCBS), Bangalore, India. Sequences were aligned using ClustalW against the *P. aeruginosa* reference strain PAO1 (NC_002516.2) to detect indels and SNPs. Mutations detected in only one strain per gene were confirmed by at least two independent sequencing experiments.

### Quantitative (qPCR)

RNA was isolated from stationary-phase cultures using the NucleoSpin RNA kit (Macherey-Nagel, 740955.50). cDNA was prepared using the RevertAid™ First Strand cDNA Synthesis Kit (Thermo Scientific, K1621). qPCR was performed with SYBR Premix Ex Taq II (Takara, RR820A) using the primers listed in Table S1 for *mexA*, *mexB*, *mexC*, *mexD*, *mexE*, *mexF*, *gyrA*, *gyrB*, *parC*, *parE* and *oprD* on an Applied Biosystems StepOnePlus™ instrument. Fold change in gene expression was calculated by the 2^(−ΔΔCT)^ method by normalizing to *rpoS* within each strain and PAO1 gene expression.

### Data analysis and statistical analysis

All final data for mutations (Table S2), antibiotic susceptibility testing (Table S3) and expression analysis (Tables S4 and S5) were analysed using Python packages Pandas, Matplotlib, NumPy, Seaborn and SciPy, and can be accessed at https://github.com/nitdmenon/Pa-MutAnalysis.git. Fisher’s exact test (two-tailed) was used to determine correlation of mutations with MDR or non-MDR groups. Significance in differential gene expression between MDR and non-MDR strains was assessed by two sided *t*-test.

### In silico modelling of SNPs

Protein sequences used from the UniProt database (https://www.uniprot.org/; accessed December 2022) and the sequences were searched against the RCSB PDB (www.rcsb.org) structural database using the NCBI BLASTp server (https://blast.ncbi.nlm.nih.gov/; accessed December 2022). Published structures with greatest sequence homology to the protein of interest were identified based on sequence identities, and detailed structure analysis was then performed in the PyMOL Molecular Graphics System, Version 2.0 (Schrödinger, LLC) including analysis of the single point substitutions. The calculated van der Waals overlap was used to select the rotamer with least steric clashes/overlap with surrounding residues to be used in post-substitution interaction analysis.

### Ethics

This study was approved by the Institutional Ethics Committee, Amrita Institute of Medical Sciences (IEC-AIMS-2019-SBT-097) and the Tata Institute for Genetics and Society (TIGS) Institutional Biosafety Committee (IBSC) review board (2019).

## Results

### The majority of P. aeruginosa clinical isolates within our sample exhibited antibiotic resistance

**Figure 1.**
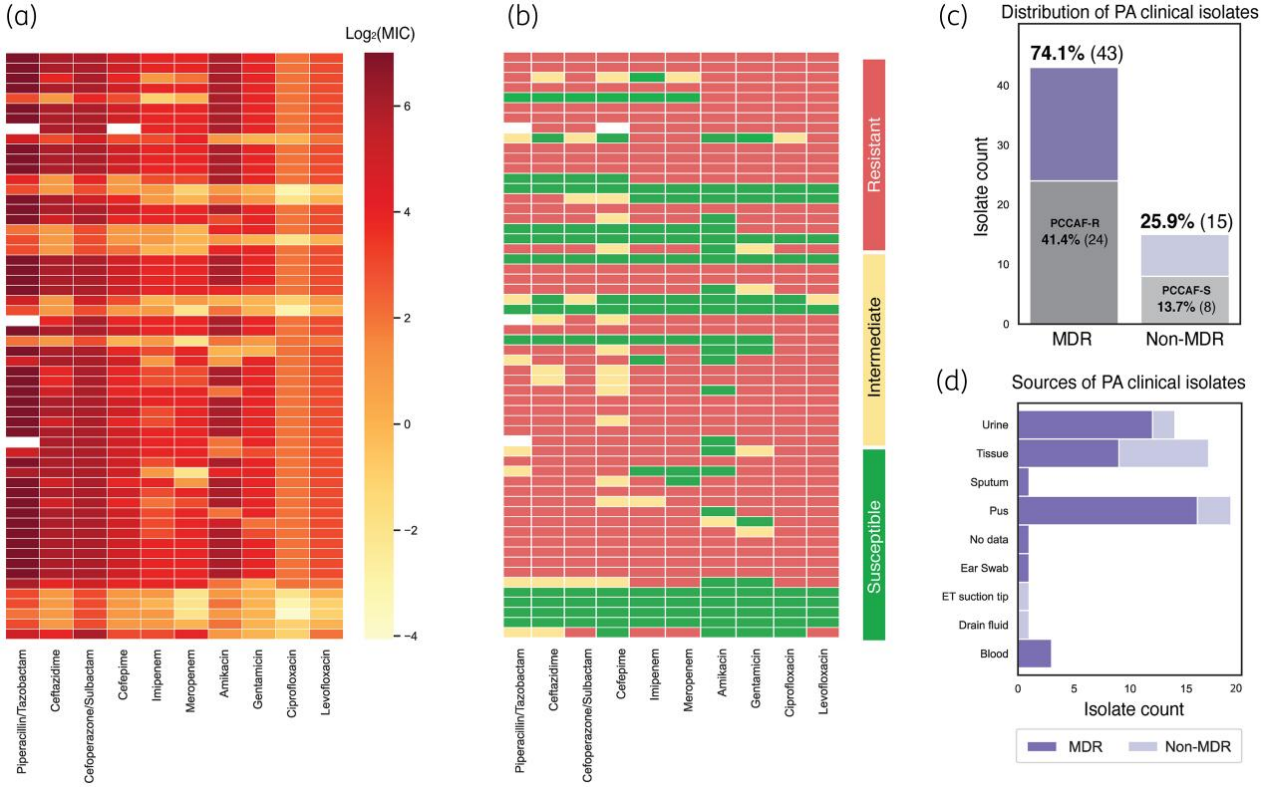
Antibiotic susceptibility testing of 58 P. aeruginosa (PA) clinical isolates from Kerala, India represented by (a) log_2_ MIC and (b) susceptibility defined by CLSI breakpoints. (c) Proportion of MDR versus non-MDR isolates based on antibiotic resistance profiles. The proportion of isolates resistant and susceptible to the entire tested antibiotic panel [PCCAF resistant (PCCAF-R) and PCCAF susceptible (PCCAF-S)] are also highlighted. (d) Distribution of MDR/non-MDR isolates by infection sources.

We obtained 58 *P. aeruginosa* isolates from hospitals and diagnostics labs in Kerala, and their susceptibility to a panel of antibiotics was assessed using the automated VITEK 2 system confirmed by disc diffusion methodology [Figure 1(a and b), Table S3].^15^ Out of these isolates, 74.1% (*n* = 43) were classified as MDR, indicating resistance to antibiotics from at least three tested classes. The remaining 25.9% (*n* = 15) were non-MDR, showing resistance to fewer than three tested classes (Figure 1c). Furthermore, 41.4% (*n* = 24) of isolates displayed resistance to the entire antibiotic panel, while 13.7% (*n* = 8) were susceptible to all test antibiotics. The antibiotic test panel included antipseudomonal penicillins, cephalosporins, carbapenems, aminoglycosides and fluoroquinolones (PCCAF). The clinical isolates were primarily obtained from pus, urine and tissue samples (Figure 1d).

### MDR clinical isolates exhibit novel mutations in regulators of MexAB-OprM and MexEF-OprN efflux pumps

**Figure 2.**
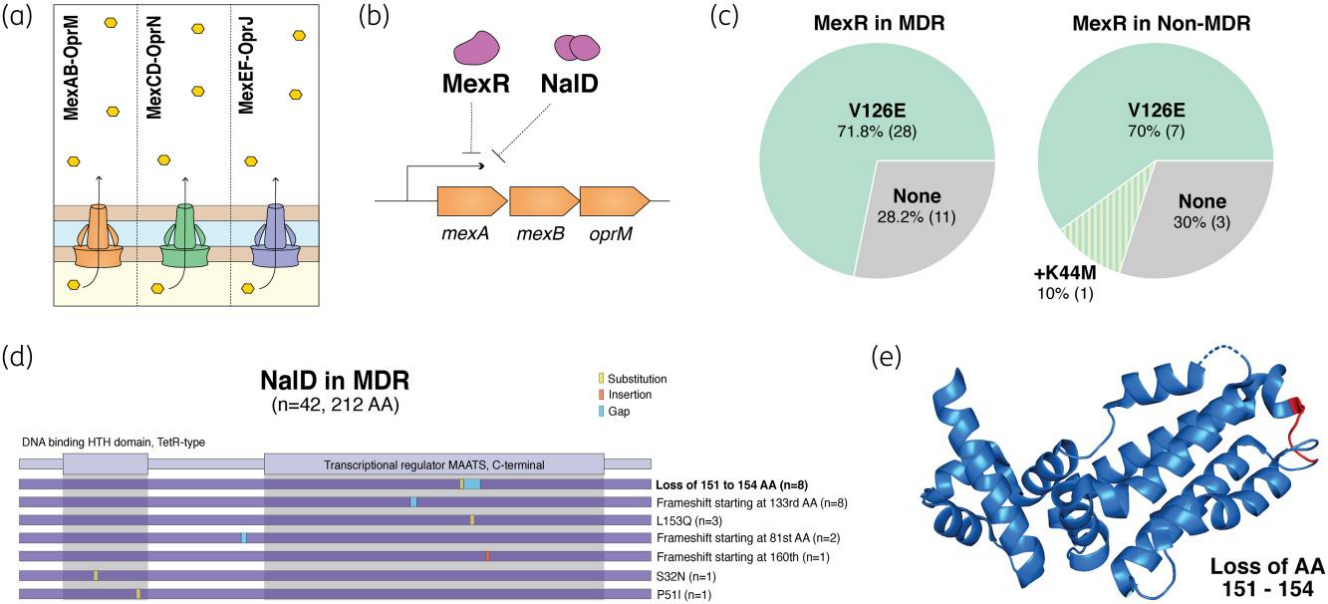
(a) Mex efflux pump systems in *P. aeruginosa* include MexAB-OprM, MexCD-OprN and MexEF-OprJ. (b) Regulation of *mexAB-oprM* expression through MexR and NalD transcriptional repressors. (c) Proportion of MexR mutations observed in screened MDR (*n* = 39) and non-MDR (*n* = 10) isolates. (d) Graphical representation of MDR-specific mutations and indels within NalD observed in a total of 24 of 42 MDR screened isolates. Motifs from the Pfam database are highlighted to showcase presence of mutations in these regions. (e) Indicates the frameshift deletion of amino acids (AA) 151–154.

The tripartite RND efflux pump systems are known to be a major contributor to multidrug resistance in *P. aeruginosa.* The MexAB-OprM, MexCD-OprJ and MexEF-OprN are the most studied of these systems (Figure 2a).^9^ Mutational analysis of antibiotic efflux pump-associated genes in MDR and non-MDR isolates revealed their significance in MDR phenotypes of regional isolates (Table 1). Sequencing of *mexR*, encoding the repressor of the *mexAB-oprM* operon (Figure 2b), identified previously reported mutations (Table S6), but only one SNP (V126E) was observed in MDR isolates compared with the reference PAO1 WT strain (Figure 2c). However, this mutation was present at similar frequencies in both MDR (71.8%) and non-MDR (70%) strains and is known not to be causative of an MDR phenotype.

**Table 1.** List of mutations found in antibiotic efflux pump-associated genes in MDR and non-MDR clinical isolates

Gene	Mutation	Amino acid change	Frequency in MDR, *n*/*N* (%)	Frequency in non-MDR, *n*/*N* (%)	*P* value	MDR specific?	MDR correlated?
*mexR*	T377A	V126G	28/39 (71.7)	7/10 (70)	1	No	No
	A131T	K44M	0/39 (0)	1/10 (10)	0.2041	No	No
*nalD*	G415T; Deletion from 452 to 462 : TGCGGCTGCAT	Loss of AAs 151 to 154	8/42 (19.0)	0/11 (0)	0.1813	Yes	No
	T397:, G398:	Frameshift starting at 133rd AA	8/42 (19.0)	0/11 (0)	0.1813	Yes	No
	T458A	L153Q	3/42 (7.1)	0/11 (0)	1	Yes	No
	G242:	frameshift starting at 81st AA	2/42 (4.8)	0/11 (0)	1	Yes	No
	476.1C	Frameshift starting at 160th	1/42 (2.4)	0/11 (0)	1	Yes	No
	G95A	S32N	1/42 (2.4)	0/11 (0)	1	Yes	No
	T151A	P51I	1/42 (2.4)	0/11 (0)	1	Yes	No
	C485A, C486G	A162E	3/42 (7.1)	1/11 (9.1)	1	No	No
	Deletion from 476 to 494:CGAGGATCGCCTCGCGCGC	Frameshift starting at 158th AA	1/42 (2.4)	1/11 (9.1)	0.3752	No	No
	G360T, C441G, A469C, A560C, T561C	E120D, D147E, M157L, D187A	0/42 (0)	1/11 (9.1)	0.2075	No	No
	G545C, Deletion from 547 to 557:CTGTTCGATCC	R182P, Frameshift from 183rd AA	0/42 (0)	1/11 (9.1)	0.2075	No	No
	None	None	12/42 (28.6)	7/11 (63.6)	0.0411	No	Yes
*mexS*	G745A	D249N	26/28 (92.9)	8/8 (100)	1	No	No
	G952A	V318I	0/28 (0)	1/8 (12.5)	0.2222	No	No
	T218C	V73A	1/28 (3.6)	0/8 (0)	1	Yes	No
	T245C, C443G, C536G, G543C	L82P, T148S, S179C, E181D	0/28 (0)	1/8 (12.5)	0.2222	No	No
	T644C	V215A	0/28 (0)	1/8 (12.5)	0.2222	No	No
	None	None	2/28 (7.1)	0/8 (0)	1	Yes	No
*mexT*	Deletion from 235 to 242 : GGCCAGCC (8 bp)	Loss of 79th and 80th AA followed by frameshift	35/35 (100)	7/10 (70)	0.0085	No	Yes
	T514A	F172I	32/35 (91.4)	10/10 (100)	1	No	No
	C178T	P60S	1/35 (2.8)	2/10 (20)	0.1195	No	No
	G119A	R40H	1/35 (2.8)	0/10 (0)	1	Yes	No
	G427A	A143T	0/35 (0)	1/10 (10)	0.2222	No	No

Fisher’s exact test (two-tailed) was performed to determine correlation of mutations with MDR or non-MDR groups. AA, amino acid.

NalD is another repressor of the *mexAB-oprM* operon, which binds downstream to the MexR binding site. Mutations in *nalD* were previously reported in clinical strains overproducing MexAB-OprM but lacking inactivating mutations in MexR.^18^ Seven distinct MDR-specific mutations in *nalD* were observed in 57.1% (24/42) screened MDR isolates (Figure 2d), and their presence was significantly correlated with multidrug resistance (*P *= 0.0411). The most common mutations included loss of amino acid residues (151–154) (19%) (Figure 2e) and frameshift mutations starting at the 133rd amino acid residue (19%). MDR-subset specific single nucleotide indels resulting in a frameshift starting at 81st (4.8%) and 160th (2.4%) residues were also observed. Unique MDR-strain associated SNPs in *nalD* resulted in amino acid substitutions such as L153Q (7.1%), S32N (2.4%) and P51I (2.4%). Although *nalD* frameshifts have been reported previously in fluoroquinolone-resistant isolates in Spain, these specific frameshift mutations are novel.^19^

**Figure 3.**
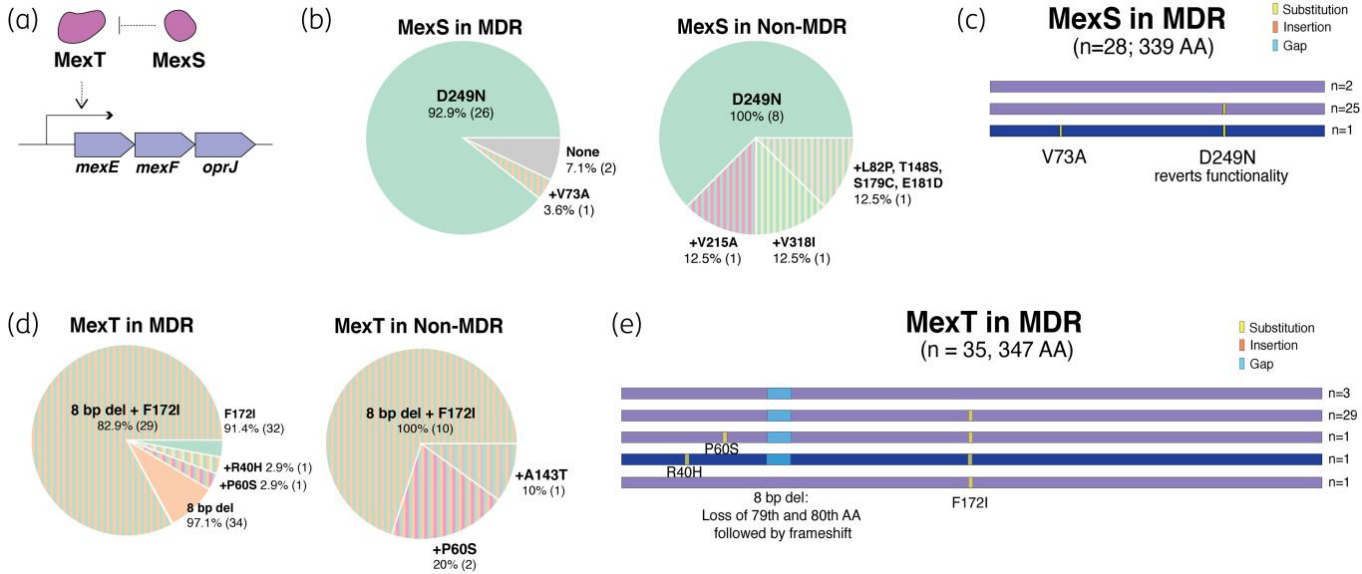
(a) MexEF-OprJ efflux pump operon regulation is mediated through activator MexT and its repressor, MexS. (b) Proportion of MexS mutations observed in screened MDR (*n* = 28) and non-MDR (*n* = 8) isolates and (c) graphical representation of observed mutations and indels within MexS in the MDR subset. Highlighted sequence refers to MDR-specific mutational profile. (d) Proportion of MexT mutations observed in screened MDR (*n* = 35) and non-MDR (*n* = 10) isolates and (e) graphical representation of observed mutations and indels within MexT in the MDR subset. The highlighted sequence refers to an MDR-specific mutational profile.

MexS is a negative regulator of *mexT* that activates the *mexEF-oprN* operon, and is overexpressed in *nfxC*-type mutants (Figure 3a). Among the screened MDR isolates, 7.1% carried the D249N loss-of-function mutation in *mexS* (Figure 3b) while non-MDR strains did not have this mutation. While other SNPs were present in both MDR and non-MDR isolates at similar frequencies, a specific V73A substitution, combined with the D249N mutation, was present in MDR isolates (Figure 3c). This mutation has previously been reported in combination with L270Q in MexS from a single clinical *nfxC*-harbouring isolate from France.^20^ Modelling studies suggest that V73A substitution disrupts a single H-bond (Figure 3d).

The functionality of MexS is dependent on MexT. A previously documented 8 bp insertion in *mexT* leads to a deleterious frameshift mutation in the WT PAO1.^21^ Conversely, an 8 bp deletion, as observed in our alignment using this reference, suggests the restoration of functional MexT and the potential overproduction of MexEF-OprN. Interestingly, 97.1% of the screened isolates had this 8 bp deletion, resulting in the loss of amino acid residues 79–80 and a frameshift. Additionally, 91.4% of the isolates harboured an SNP resulting in an F172I substitution in MexT (Figure 3e). These mutations were commonly found together, but as they were also present in 100% of the screened non-MDR isolates, they are unlikely to be associated with multidrug resistance. The only mutation specific to the MDR group was an SNP leading to an R40H substitution (Figure 3f).

### Increased gene expression of mexA in clinical isolates resistant to antipseudomonal PCCAF

**Figure 4.**
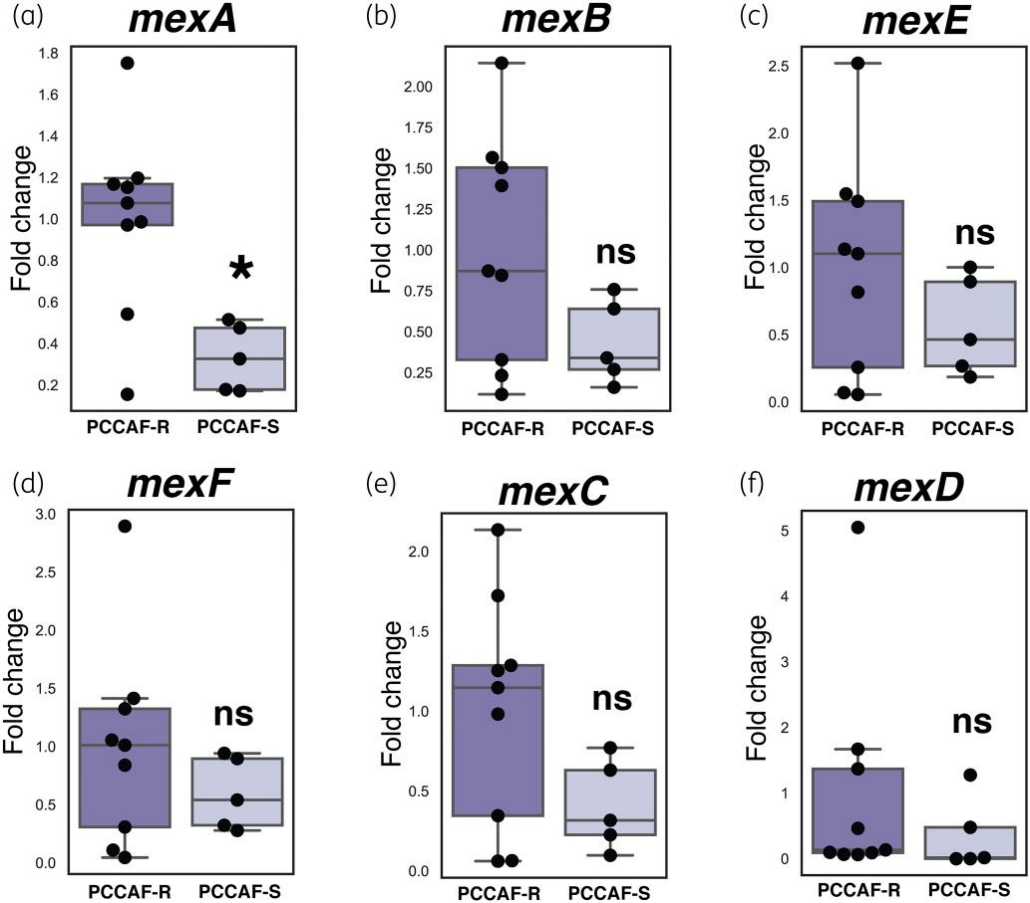
Differential gene expression of MexAB-OprM, MexEF-OprN and MexCD-OprJ RNS efflux systems in PCCAF-resistant (PCCAF-R) and PCCAF-susceptible (PCCAF-S) isolates. Differential gene expression of efflux pump genes (a) *mexA*, (b) *mexB*, (c) *mexE*, (d) *mexF*, (e) *mexC* and (f) *mexD* in a subset of nine PCCAF-R and five PCCAF-S isolates. Values are normalized to average PCCAF-R isolate gene expression. Two sided t-test was performed to determine statistical significance, *P < 0.05.

Mutations affecting the MexAB-OprM and MexEF-OprD efflux pump systems are known to contribute to resistance against multiple classes of antibiotics including quinolones, macrolides, novobiocin, chloramphenicol, tetracyclines, lincomycin and β-lactams.^22^ Since we identified mutations exclusively in MDR strains affecting the MexAB-OprM and MexEF-OprD efflux pump systems, we were therefore interested in understanding if there were alterations in expression of these efflux pumps in isolates that were resistant versus susceptible to all tested antibiotics. Correlating with the mutational studies of *nalD*, we observed significantly higher expression of *mexA* in a subset of eight PCCAF-resistant strains compared with five PCCAF-susceptible isolates (Figure 4a). Although there was a trend of increased expression in *mexB*, *mexE*, *mexF*, *mexC and mexD* in PCCAF-resistant isolates, the differences were not statistically significant [Figure 4(b–f)].

### Mutations in QRDRs of fluoroquinolone determinants are significantly correlated with MDR phenotype

**Figure 5.**
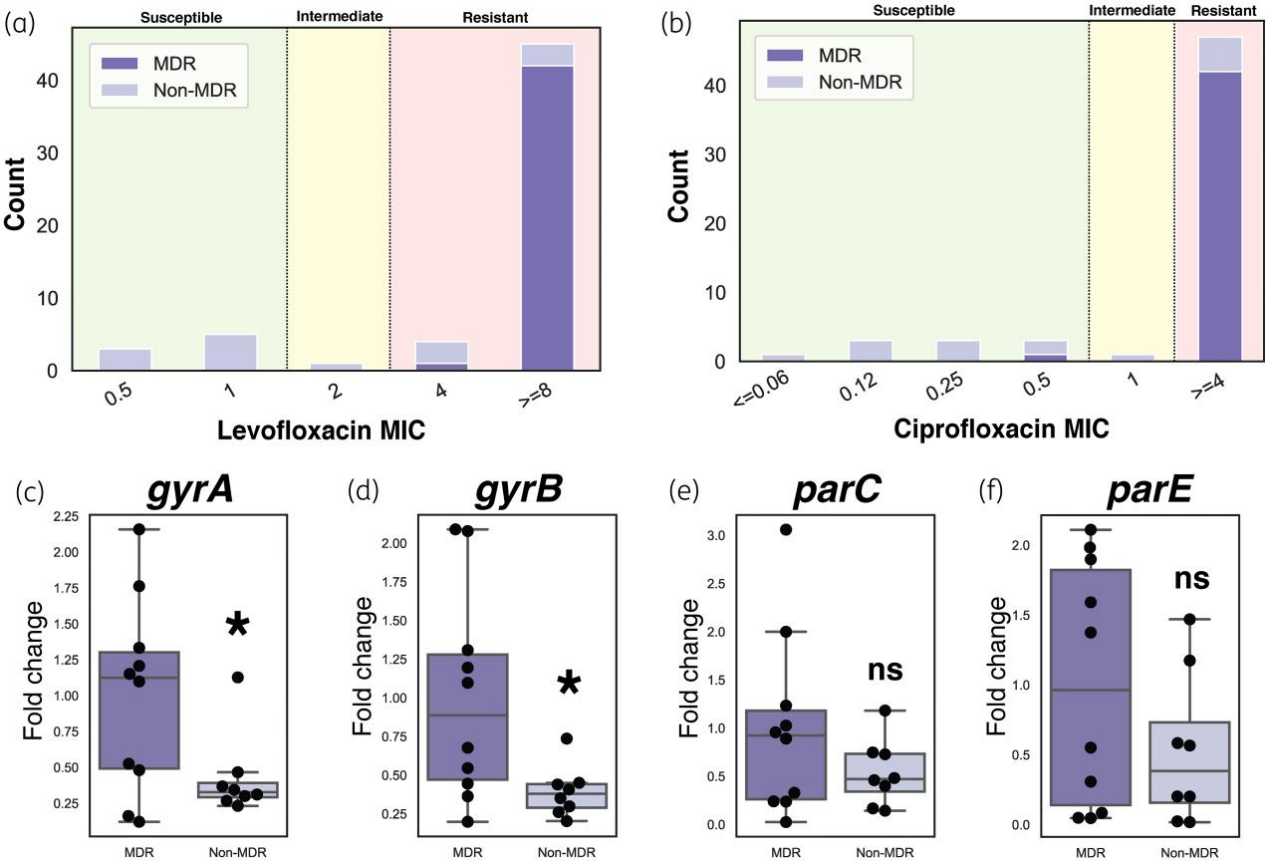
Fluoroquinolone resistance across MDR and non-MDR isolates. Distribution of entire panel of MDR and non-MDR strains across (a) ciprofloxacin and (b) levofloxacin MIC values with ranges denoted as susceptible, intermediate and resistant as per CLSI breakpoints. Relative expression of fluoroquinolone-resistance genes (c) *gyrA*, (d) *gyrB*, (e) *parC* and (f) *parE* in MDR and non-MDR isolates. Values are normalized to average MDR isolate gene expression. Two sided *t*-test was performed to determine statistical significance; *P < 0.05.

All regional MDR clinical isolates of *P. aeruginosa* exhibited resistance to fluoroquinolones, with 100% showing resistance to levofloxacin (Figure 5a) and 97.6% showing resistance to ciprofloxacin (Figure 5b). Fluoroquinolone resistance occurs primarily through mutations in antibiotic targets, namely DNA gyrase (*gyrA* and *gyrB*) and DNA topoisomerase IV (*parC* and *parE*), but could also be due to their increased expression. MDR clinical isolates exhibited increased expression of DNA gyrase subunits *gyrA* and *gyrB* [Figure 5(c–f)].

**Figure 6.**
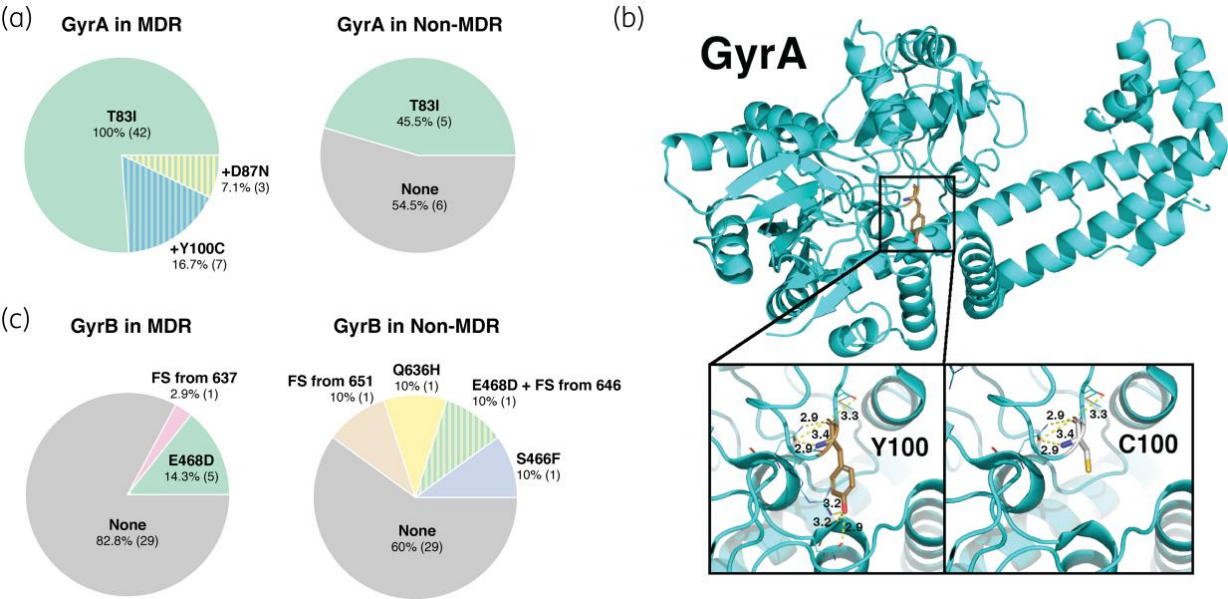
Mutational analysis of DNA gyrase. (a) Proportion of GyrA mutations in MDR (*n* = 42) and non-MDR (*n* = 11) clinical isolates and (b) modelling of the MDR-specific GyrA Y100C amino acid substitution due to A299G and SNP in MDR isolates. (c) GyrB mutations in MDR (*n* = 35) and non-MDR (*n* = 10) clinical isolates. FS, frameshift.

Additionally, sequencing of the QRDR of *gyrA* and *gyrB* genes revealed conserved mutations in the MDR isolates (Table 2).^23^ Mutations in GyrA were significantly associated with the MDR phenotype (*P* < 0.0001). A T83I amino acid substitution within the QRDR region of GyrA was present in 100% of screened MDR strains (Figure 6a) compared with 45.5% of non-MDR strains (*P* < 0.0001). Notably, the presence of a secondary mutation, either Y100C or D87N, was exclusively observed in 23.8% MDR isolates. The UniProt database entry for *P. aeruginosa* GyrA (P48372) protein sequence was used to search the RCSB PDB database, which identified the gyrase crystal structure from *Escherichia coli* resolved to 3.30 Å (PDBID: 4TMA) has a sequence identity of 76%. Based on the *E. coli* structure, *P. aeruginosa* Y100 is expected to reside within a loop adjacent to an α-helix. Both the Y100 and loop residues form hydrogen-bonding interactions with this neighbouring α-helix. A Y100C mutation is expected to result in the loss of two hydrogen-bonding interactions that had been formed by the Y100 hydroxyl group (Figure 6b). The D87N substitution is a more conservative mutation and thus similar interactions with the protein structure are likely maintained following mutagenesis from D to N. However, a recent report suggests that the combination of T83I with D87N mutation may impact the binding interactions of quinolone drugs to the GyrA subunit.^24^ Based on docking studies, it was speculated that the D87N mutation contributed to quinolone resistance when acting in combination with the primary T83I mutation.^24^ Although GyrA T83I and D87N are known fluoroquinolone-resistance SNPs, Y100C is novel and has not been reported in the literature before.

**Table 2.** List of mutations found in MDR and non-MDR isolates in fluoroquinolone resistance-associated genes

Gene	Mutation	Amino acid change	Frequency in MDR, *n*/*N* (%)	Frequency in non-MDR, *n*/*N* (%)	*P* value	MDR specific?	MDR correlated?
*gyrA*	C248T	T83I	42/42 (100)	5/11 (45.5)	<0.0001	No	Yes
	A299G	Y100C	7/42 (16.7)	0/11 (0)	0.3221	Yes	No
	G259A	D87N	3/42 (7.1)	0/11 (0)	1	Yes	No
	None	None	0/42 (0)	6/11 (54.5)	<0.0001	No	Yes
*gyrB*	G1404C/T	E468D	5/35 (14.3)	1/10 (10)	1	No	No
	C1397T	S466F	0/35 (0)	1/10 (10)	0.2222	No	No
	G1909:	Frameshift from 637th AA and premature stop codon at 639th position	1/35 (2.8)	0/10 (0)	1	Yes	No
	1935.1C insertion downstream of QRDR region	Frameshift from 646th AA	0/35 (0)	1/10 (10)	0.2222	No	No
	G1908T	Q636H	0/35 (0)	1/10 (10)	0.2222	No	No
	G1951:	Frameshift from 651st AA	0/35 (0)	1/10 (10)	0.2222	No	No
	None	None	29/35 (82.9)	6/10 (60)	0.1937	No	No
*parC*	C260T/G	S87L	35/40 (87.5)	4/10 (40)	0.004	No	Yes
	G262C	A88P	2/40 (5.0)	0/10 (0)	1	Yes	No
	142.1G	Frameshift from 48th AA	1/40 (2.5)	0/10 (0)	1	Yes	No
	C357:	K120S	0/40 (0)	1/10 (10)	0.2	No	No
	None	None	5/40 (12.5)	5/10 (50)	0.0181	No	Yes
*parE*	A1369G	S457G	2/35 (5.7)	0/10 (0)	1	Yes	No
	A1376T	E459V	1/35 (2.8)	0/10 (0)	1	Yes	No
	C1371G	S457R	1/35 (2.8)	1/10 (10)	0.399	No	No
	C1599A	D533E	1/35 (2.8)	3/10 (30)	0.0296	No	Yes
	G1255A	D419N	1/35 (2.8)	0/10 (0)	1	Yes	No
	None	None	29/35 (82.9)	6/10 (60)	0.0026	No	Yes

Fisher’s exact test (two-tailed) was performed to determine correlation of mutations with MDR or non-MDR groups.

Mutations in GyrB were found in 17.4% of the MDR isolates and 40% of the non-MDR isolates (Figure 6d). The E468D mutation was exclusive to the MDR group, except for its occurrence in a single fluoroquinolone-resistant non-MDR strain. Additionally, a premature stop codon resulting in a truncated 639 amino acid protein was seen in one MDR isolate. The E468D mutation has been previously reported in *exoU/exoS*-harbouring isolates from anterior eye infections, microbial keratitis and the lungs of cystic fibrosis patients.^25,26^ Modelling of this amino acid substitution suggests that its impact may not be significant, as it exhibits similar H-bonding patterns (Figure 6e).

**Figure 7.**
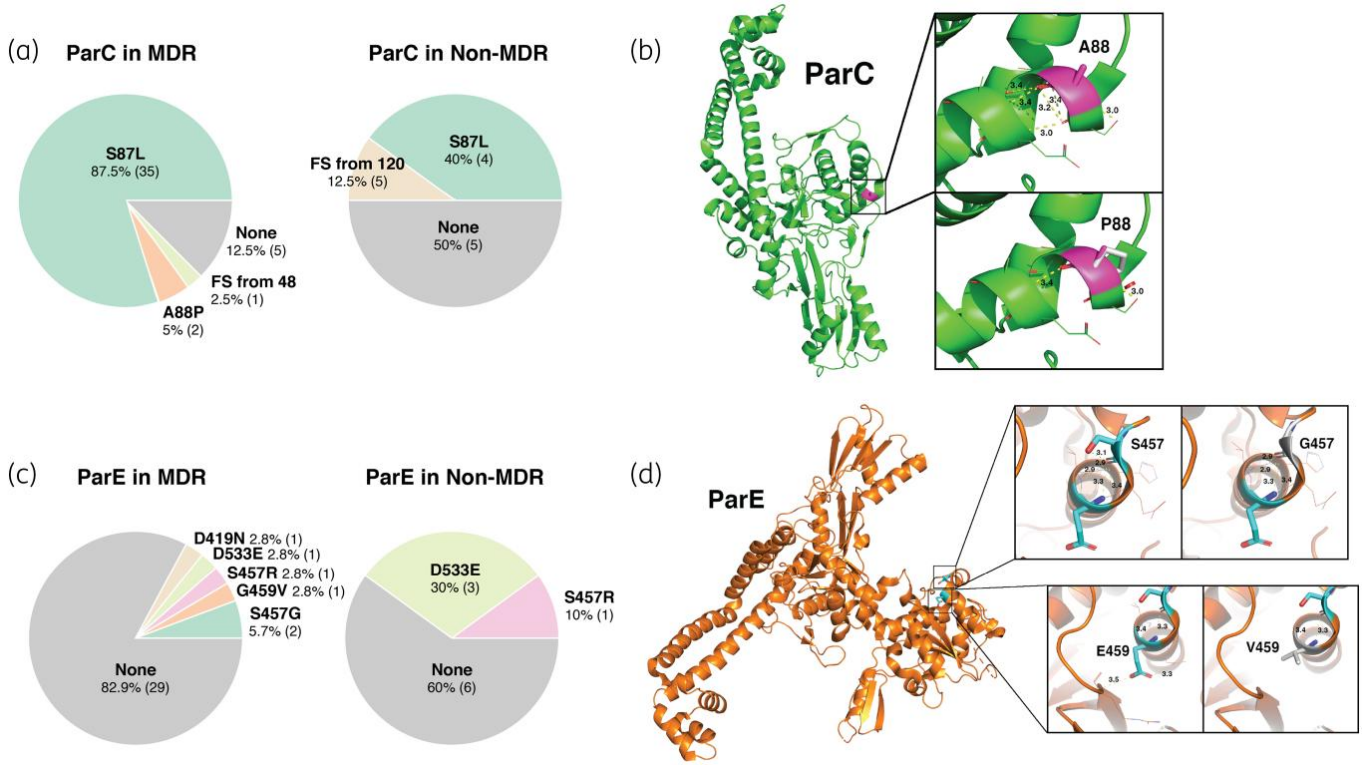
Mutational analysis of DNA topoisomerase IV. (a) ParC mutations in MDR (*n* = 40) and non-MDR (*n* = 10) isolates and (b) modelling of the MDR-specific ParC A88P amino acid substitution due to G262C SNP in MDR isolates. (c) ParE mutations in MDR (*n* = 35) and non-MDR (*n* = 10) clinical isolates and (d) modelling of MDR-specific ParE amino acid substitutions E459V and S457G due to SNPs in MDR isolates. FS, frameshift.

The presence of a mutation in the QRDR region of ParC, the GyrA homologue of DNA topoisomerase IV, was significantly associated with the MDR phenotype (*P *= 0.0181). The S87L mutation was predominantly found in the MDR group (*P *= 0.004) as it was present in 87.5% of MDR strains compared with only 40% of tested non-MDR strains (Figure 7a). Exclusive secondary mutations, A88P and a frameshift after the 46th amino acid residue, were seen in a total of 7.5% of MDR isolates. The *P. aeruginosa parC* sequence has 88% sequence identity to the N-terminal fragment of DNA topoisomerase IV subunit A from *Psuedomonas putida* (PDB code: 6BQ9) that has a crystal structure determined at 2.55 Å. This crystal structure indicates that *P. aeruginosa* residue A88 likely lies within an α-helix and forms hydrogen-bonding interactions within the α-helical turn, as depicted in Figure 7(b); mutation from alanine to proline at this position is expected to disrupt the α-helix and disrupt the secondary structure in this region of DNA topoisomerase IV subunit A. Three ParE mutations (D419E, S457G and E459V) previously reported in fluoroquinolone isolates from Taiwan were uniquely observed in the MDR group (Figure 7c).^27^ The D533E mutation showed a significant correlation with non-MDR isolates, suggesting the WT sequence may be associated with the MDR phenotype. *P. aeruginosa* topoisomerase IV subunit B has 75% sequence homology to the *Acinetobacter baumannii* topoisomerase IV that has a crystal structure determined to 3.25 Å (PDB code: 2XKK). Based on this homology, single point substitutions S457G and E459V in *P. aeruginosa* are depicted in Figure 7(d). The S457G substitution would result in the loss of a single hydrogen-bond interaction formed between the serine hydroxyl group and the peptide backbone that may contribute to the stability of the α-helix (Figure 7d). The E459V substitution is non-conservative and introduces a hydrophobic residue in a region previously containing charged interactions. This likely results in a loss of hydrogen-bonding interactions with both the α-helical residue, D462, and nearby β-sheet residue, R439, potentially destabilizing the secondary structure packing in this region (Figure 7d).

### MDR strains exhibit carbapenem resistance with concomitant genomic and expression variations

**Figure 8.**
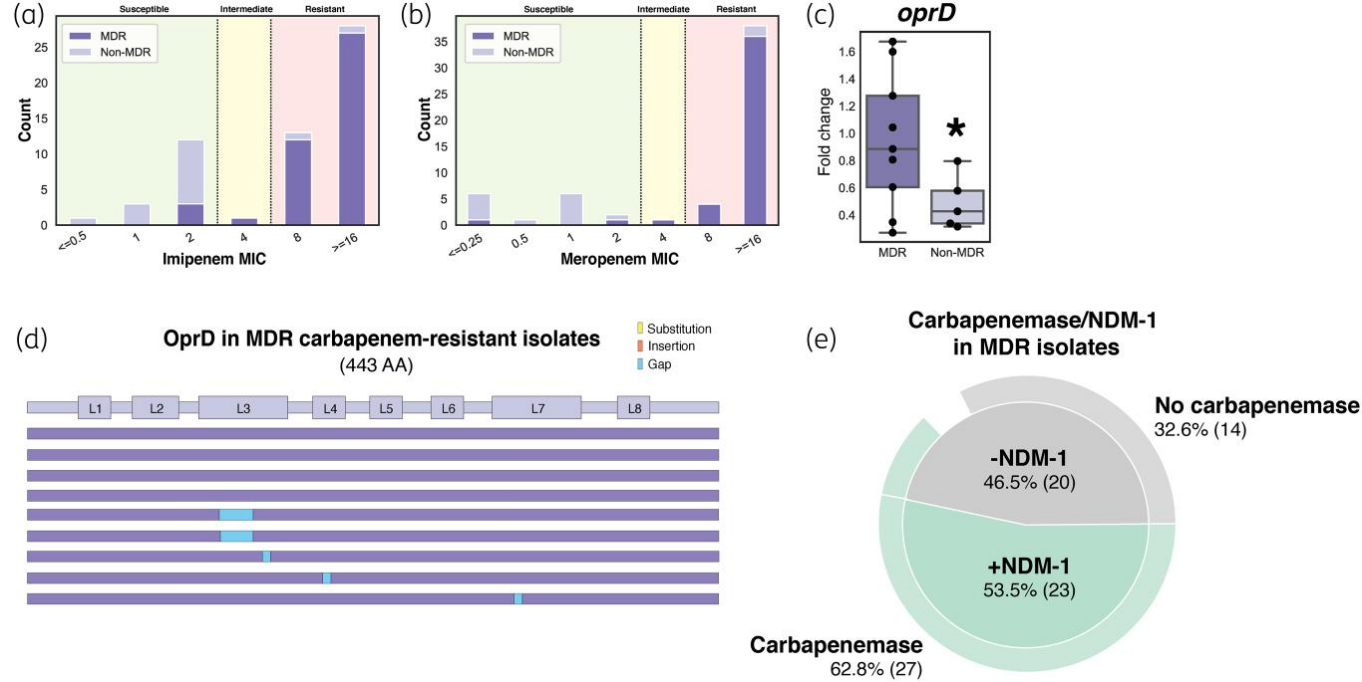
Distribution of MDR and non-MDR strains across (a) imipenem and (b) meropenem MIC values with ranges denoted as susceptible, intermediate and resistant as per CLSI breakpoints. (c) Relative expression of *oprD* in carbapenem-resistant MDR (*n* = 9) and non-MDR (*n* = 5) isolates. Values are normalized to average MDR isolate gene expression. Two sided *t*-test was performed to determine statistical significance; *P < 0.05. (d) *oprD* mutations in the nine carbapenem-resistant MDR isolates. (e) Pie chart showcasing the proportion of MDR isolates with carbapenemase activity as determined by mCIM assay and MDR isolates containing *bla*_NDM-1_.

Among the 43 screened MDR strains, 90.7% (*n* = 39) were imipenem resistant and 93.0% (*n* = 40) were meropenem resistant [Figure 8(a and b)]. The loss of OprD, a carbapenem entry porin, is a common adaptive mutation associated with carbapenem-resistant strains of *P. aeruginosa.* Interestingly, the expression of *oprD* was significantly higher in MDR, carbapenem-resistant strains compared with the non-MDR, carbapenem-susceptible subset of isolates (Figure 8c).

To explore the potential presence of mutations leading to the loss of OprD function, we examined nine MDR, carbapenem-resistant isolates and four non-MDR clinical isolates of *P. aeruginosa*, for mutations in OprD (Figure 8d). Of these MDR strains, deletions were present in loop 3 (33.3%), loop 4 (11.1%) and loop 7 (11.1%). None of the screened non-MDR isolates showed significant mutations or deletions in the *oprD* gene.

Due to the inability to attribute resistance mechanisms to the majority of carbapenem-resistant regional isolates through mutational or expression analysis, we investigated the possible involvement of antibiotic modification in conferring resistance. Screening for carbapenemase activity and presence of *bla*_NDM-1_ was conducted. A significant proportion of the 57 screened *P. aeruginosa* clinical isolates exhibited carbapenemase activity (47.36%) and carried *bla*_NDM-1_ (40.35%). These findings were strongly correlated with MDR phenotype (*P *< 0.0001 and 0.0003, respectively) (Table 3). Within the MDR subset, there was a high correlation between carbapenemase activity and the presence of NDM-1 (*P *= 0.0001), as 23 out of the 27 carbapenemase-positive isolates also tested positive for NDM-1.

**Table 3.** Carbapenemase and presence of NDM-1 in clinical *P. aeruginosa* isolates

		Number of isolates	Percentage	*P* value (MDR v. non-MDR)
Carbapenemase	Total (57)	27	47.36	<0.0001
	MDR (43)	27	62.79	
	Non-MDR (14)	0	0	
No carbapenemase	Total (57)	28	49.12	
	MDR (43)	14	32.56	
	Non-MDR (14)	14	100	
No data	Total (57)	2	3.51	
	MDR (43)	2	4.65	
	Non-MDR (14)	0	0	
*bla*_NDM-1_ positive	Total (57)	23	40.35	0.0003
	MDR (43)	23	53.49	
	Non-MDR (14)	0	0	
*bla*_NDM-1_ negative	Total (57)	34	59.64	
	MDR (43)	20	46.51	
	Non-MDR (14)	14	100	

## Discussion

In India, the rates of AMR in *P. aeruginosa* are alarmingly high, yet data that disclose these statistics across the country are limited. Furthermore, there is a scarcity of reports that examine the presence of genetic determinants, mutations and differential gene expression related to resistance-associated genes in MDR clinical isolates. Therefore, a deeper understanding of regional resistance mechanisms is critical for developing lasting solutions at both the local and global levels.

Through an analysis of a convenience sample—a library of 58 *P. aeruginosa* clinical isolates—from the Kerala region, our aim was to identify mutations and relative expression profiles that could be specifically associated with MDR, rather than being simply representative of regional clonal groups. We observed mutations present in both MDR and non-MDR isolates, as well as mutations unique to either group. This highlights the importance of using the correct parent strain for comparative studies to ensure an accurate understanding of the selection of resistance mechanisms. For example, although previous reports have described inactivating mutations in MexR in antibiotic-resistant strains in India, our findings indicate that these mutations were not specific to MDR strains.^28^ This is consistent with global studies suggesting that such substitutions are maintained within phylogenetic lineages rather than being MDR specific.^29,30^

More than half of the screened MDR strains had MDR-specific NalD mutations, including novel mutations such as L153Q, S32N and P51I. Prior studies have shown that mutations in the 10th helix of NalD can disrupt its dimerization repressor activity, predictive of aztreonam resistance.^31^ This suggests that this mechanism of resistance may be prevalent in regional isolates. Additionally, we observed significantly increased expression of the MexAB-OprM genes, along with trends of increased expression of other efflux pumps in MDR isolates. Further analysis with larger datasets is needed to confirm the significance of these trends.

It is noteworthy that all MDR clinical isolates in our study were resistant to levofloxacin and/or ciprofloxacin, which aligns with findings from other research groups globally.^32^ This suggests that mutations associated with fluoroquinolone resistance, such as GyrA T83I and ParC S87L, could serve as prominent biomarkers for MDR in *P. aeruginosa* infections that are challenging to treat. Interestingly, only the expression of *gyrA* and *gyrB* was significantly higher in MDR isolates, indicating that in regional isolates, the primary target of fluoroquinolones is likely mediated through DNA gyrase. These bacterial populations may allocate resources to constitutively increase the production of target enzymes, even in the absence of antibiotics.

Previous reports have highlighted the loss of function of entry porins as a major contributor to carbapenem resistance in *P. aeruginosa*.^33^ We observed that approximately half of the screened MDR isolates harboured inactivating deletions in OprD. However, we also observed an unexpected increase in *oprD* expression in MDR strains compared with non-MDR isolates, suggesting a potential compensatory mechanism. Importantly, all MDR isolates within the carbapenem-resistance qPCR study carried the *bla*_NDM-1_ gene encoding NDM-1. This gene was also present in 23/43 of the screened MDR isolates (53.5%), highlighting its widespread role in resistance phenotypes in the region.

To ascertain causation of the MDR phenotype, we conducted molecular modelling of multiple identified SNPs, including those in GyrA, ParC and ParE, some of which revealed changes in protein conformation that could impact activity. These newly identified genetic determinants of resistance in regional clinical isolates of *P. aeruginosa* underscore the prevalence of resistance across different antibiotic classes and provide insight into potential resistance mechanisms adopted by the pathogen.

Expanding these studies to include larger numbers of isolates from multiple regions with additional screening of various antibiotic classes would further the knowledge on the molecular complexities and epidemiology of antibiotic resistance. Furthermore, this could provide valuable information for identification and diagnosis of infections with specific resistance phenotypes and guide the development of therapeutic regimens to improve clinical outcomes.

## Supplementary Material

dlae001_Supplementary_Data

## Supplementary data

Tables S1 to S3 are available as Supplementary data at *JAC-AMR* Online.
